# Perceptions of attitudes toward statistics among medical undergraduates: insights from a regional medical college in China

**DOI:** 10.1186/s12909-024-05600-1

**Published:** 2024-05-27

**Authors:** Yupeng Guo, Shengzhong Rong, Jing Dong, Tao Ji, Yingying Niu, Hongjun Guan

**Affiliations:** Department of Epidemiology and Medical Statistics, School of Public Health of Mudanjiang Medical University, Tongxiang Road, Aimin District, Mudanjiang City, Heilongjiang Province 157011 China

**Keywords:** Statistics, Attitudes towards statistics, Medical undergraduate

## Abstract

**Background:**

Among Chinese medical students, medical statistics is often perceived as a formidable subject. While existing research has explored the attitudes of Chinese postgraduate medical students towards statistics and its impact on academic performance, there is a scarcity of studies examining the attitudes of Chinese medical undergraduates on this subject. This study endeavors to scrutinize the attitudes of Chinese medical undergraduates towards statistics, assessing their ramifications on learning achievements, and delving into the influence of demographic factors.

**Methods:**

1266 medical undergraduates participated in this study, completing a questionnaire that included SATS-36 and additional queries. Furthermore, an examination was administered at the end of the medical statistics course. The analysis encompassed the SATS score and exam scores, examining both the overall participant population and specific demographic subgroups.

**Results:**

Undergraduate medical students generally exhibit a favorable disposition towards statistics concerning Affect, Cognitive Competence, and Value components, yet harbor less favorable sentiments regarding the Difficulty component of SATS-36, aligning with previous research findings. In comparison to their postgraduate counterparts, undergraduates display heightened enthusiasm for medical statistics. However, they demonstrate a lower cognitive capacity in statistics and tend to underestimate both the value and difficulty of learning statistics. Despite these disparities, undergraduate medical students express a genuine interest in statistics and exhibit a strong dedication to mastering the subject. It is noteworthy that students’ attitudes toward statistics may be influenced by their major and gender. Additionally, there exists a statistically significant positive correlation between learning achievement and the Affect, Cognitive Competence, Value, Interest, and Effort components of the SATS-36, while a negative correlation is observed with the Difficulty component.

**Conclusion:**

Educators should carefully consider the influence of attitudes toward statistics, especially the variations observed among majors and genders when formulating strategies and curricula to enhance medical statistics education.

## Introduction

Medical statistics is a crucial branch of statistics that encompasses various aspects of medical and biological research, including experiment and trial design, data collection, analysis, and representation. Around the World, medical students are required to take medical statistics courses to acquire the necessary skills and abilities in statistics. These skills play a pivotal role in the professional development of medical practitioners [1–3]. Over the past few decades, with the emergence of Evidence-based medicine (EBM), the knowledge and skills in medical statistics have become increasingly important for future doctors [2].

Considering the importance of medical statistics, numerous studies have revealed that medical students generally view the subject as challenging and experience anxiety when learning about it. Compared to other disciplines in medical training, the majority of students state that the medical statistics course is more challenging. Researchers have attributed this phenomenon to the mathematical properties of statistics, but numerous studies have shown that students’ attitudes toward statistics are important when it comes to learning statistics. Students generally recognize the value and usefulness of statistics, but they also find it difficult to learn. Positive attitudes are positively correlated with course achievement [4–12], and additional factors such as demographics [13], educational backgrounds [14], and mathematical foundations [4] may also have an impact on students’ attitudes toward statistics.

In China, medical statistics is a mandatory subject for both undergraduate and postgraduate medical students. A study by Zhang et al. [4] investigated the attitudes of medical postgraduates toward medical statistics using the Survey of Attitudes Toward Statistics (SATS) [15]. The findings revealed that Chinese medical students generally held positive attitudes toward statistics, although they perceived medical statistics as a challenging subject. Despite positive correlations between course achievement and attitudes towards medical statistics, students’ attitudes experienced negative changes after completing a statistics course. It’s noteworthy that Zhang et al.‘s study focused on postgraduates in an elite medical college, which is nationally renowned for its academic excellence, leaving a gap in understanding the attitudes of undergraduate medical students in regional medical colleges, which represents the non-renowned and ordinary majority. Compared with the students in elite medical colleges, these medical undergraduates enrolled in regional medical colleges generally demonstrate lower scores in the National College Entrance Examination (the Gaokao), suggesting they may have different capabilities and habits in learning science and technology, so it is necessary and meaningful to find out what attitude they hold towards medical statistics and whether their attitude is different from Zhang et al.‘s results. To address this gap, we conducted a cross-sectional survey in a typical medical college in northeastern China, examining the attitudes of undergraduate medical students towards medical statistics and exploring influencing factors, as well as the association between attitudes and course achievement. The results could contribute to alleviating undergraduates’ anxiety about learning medical statistics and have implications for curriculum design and delivery methods in the class.

## Methods

### Participants

The investigation took place at Mudanjiang Medical University, a regional medical institution situated in Heilongjiang Province, China, in April 2023. The study targeted undergraduate students enrolled in clinical medicine, dentistry, radiology, or nursing majors, specifically those in their second or third academic years. Notably, Chinese students pursuing clinical medicine, dentistry, and radiology undergo a five-year medical training program, culminating in the attainment of a medical bachelor’s degree. Conversely, nursing majors follow a four-year medical training program, leading to the award of a nursing bachelor’s degree. Due to the students with medical degrees and the students with nursing degrees having different characteristics and career prospects, to facilitate result interpretation, we categorized participants into medical undergraduates and nursing undergraduates. Medical statistics was a mandatory course for all the participants of our study, they were mandated to undertake the same medical statistics course. The curriculum of this course encompasses various topics such as descriptive statistics, probability distribution of random variables (normal distribution, t distribution, F distribution, and Chi-square distribution), confidence intervals for mean, hypothesis testing (t-test, analysis of variance, Chi-square test), nonparametric statistics, linear correlation and regression, logistics regression, general experimental design, and the application of SPSS software. Prior to their involvement, participants were thoroughly briefed on the study’s objectives and procedures, and their informed consent was obtained through signed approval documents. Although Chinese law does not mandate ethical approval for this type of study, ethical approval for this study was granted by the School of Public Health of Mudanjiang Medical University.

### Instruments

We employed the SATS-36 scale, developed by Schau [15], to assess students’ attitudes toward medical statistics. The initial version of SATS, termed SATS-28, comprised 28 7-point Likert-type scale items designed to measure four attitude components: Affect (6 items, reflecting students’ emotions regarding statistics), Cognitive Competence (6 items, gauging students’ attitudes toward their intellectual knowledge and skills applied to statistics), Value (9 items, assessing students’ perspectives on the usefulness, relevance, and worth of statistics in personal and professional contexts), and Difficulty (7 items, capturing students’ perceptions of the difficulty of statistics as a subject). Subsequently, Schau [16] introduced 8 additional items to measure two more attitude components: Interest (4 items, evaluating students’ interest in statistics) and Effort (4 items, quantifying the effort students invest in learning statistics), resulting in the formation of SATS-36. Each item was scored on a scale where higher scores indicated more positive attitudes (1 = “strongly disagree,” 4 = “neither disagree nor agree,” 7 = “strongly agree”). The component scores were computed as the mean of the item scores constituting the respective components. In instances where items featured negative wording, scores were reversed in accordance with the SATS-36 guidelines (e.g., 1 replaced by 7). The SATS-36 scale demonstrated favorable internal consistency. Schau [16] and other researchers [6, 7, 17]consistently reported high Cronbach’s alpha coefficient values for the six attitude components of SATS-36: Affect (0.80 to 0.89), Cognitive Competence (0.77 to 0.88), Value (0.74 to 0.90), Difficulty (0.64 to 0.81), Interest (0.85 to 0.88), and Effort (0.67 to 0.85), meaning the scale has good internal consistency and validity as a measurement instrument. Additionally, many studies have confirmed that SATS-36 is a cross-cultural tool that has been previously validated in different languages [18, 6, 8, 12]. In this study, we translated the scale into a Chinese version. Before the formal survey, we invited 20 students to test whether our Chinese version was clear, understandable, and acceptable. The result of the test showed that our Chinese version had good face validity.

### Investigation process

The survey utilized the Tencent questionnaire web system for data collection. Participants were instructed to complete an SATS-36 questionnaire and additional inquiries at the beginning of the course. The course was completed in 8–10 weeks. One week after the course’s end, a standard course examination was administered by the college administration to evaluate the participants’ academic performance. All the participants had to take this course examination. This closed-book standard examination was designed by the teachers of lessons and based on quantitative criteria, grading out of 100 points and comprised 40 single-choice questions (40 items, 1 point each), 10 fill-in-the-blank questions (10 blanks, 1 point each), 10 definition questions (5 items, 2 points each), and 40 points of calculation questions (4–5 items, encompassing tasks such as calculating confidence intervals and hypothesis testing). In accordance with Chinese academic norms and the college administration, students would receive academic credits upon scoring above 60 points. Since all the questions of the examination were developed according to the content of the course, examination scores could be used to evaluate the participants’ academic performance. To investigate the influence of students’ attitudes on course achievements, the SATS-36 scores would be subjected to analysis alongside the examination scores. Furthermore, demographic information, such as age, gender, and major, would be considered in the analysis.

### Statistical analysis

All statistical analyses were performed using R version 4.2.3. The demographic characteristics of participants were summarized using counts and frequencies across categories. Descriptive statistics, including mean, median, and standard deviation (SD), were employed to characterize SATS and examination scores for both the overall sample and various subgroups. Given the non-normal distribution of most SATS and examination scores, statistical comparisons among demographic factors were conducted using Wilcoxon tests. Spearman correlation coefficients were computed to investigate associations between SATS scores and examination scores, both overall and within demographic subgroups. All statistical tests were two-tailed, and significance was established at P-value ≤ 0.05.

## Results

### Participants’ demographics

The survey garnered responses from 1266 participants, with an average age of 20.65 years (Median = 21, SD = 0.92, range 18–25). Females constituted 66.1% of the participants, aligning with the typical gender distribution in Chinese medical colleges. The distribution of participants across majors appeared rational, mirroring the real-world scenario. Detailed demographic characteristics are presented in Table 1.

**Table 1:** Participants’ demographic characteristics

Characteristics	Count	Percent (%)
**Age**		
< 21	591	46.7
>=21	675	53.3
**Gender**		
Female	837	66.1
Male	429	33.9
**Major**		
Clinical medicine	578	45.7
Dentistry	27	2.1
Radiology	583	46.1
Nursing	78	6.2
Medical undergraduate	1188	93.8
Nursing undergraduate	78	6.2

### SATS scores and course achievement

The Cronbach’s α coefficient for our study was 0.71, indicating a high level of consistency in our results. Table 2 provides an overview of the mean, median, and standard deviation (SD) values for both SATS scores and examination scores. Our findings revealed predominantly positive sentiments among students regarding medical statistics, with a mean score of 4.55 for the Affect component. Participants expressed confidence in their intellectual abilities and skills to grasp medical statistics, as evidenced by a mean score of 4.63 on the Cognitive Competence Component. Recognizing the value of medical statistics in their future careers, students recorded a mean score of 5.27 on the Value component, and they demonstrated an interest in the subject with a mean score of 4.88 on the Interest component. Notably, participants displayed a strong willingness to exert additional effort in learning medical statistics, as reflected in a mean score of 6.07 on the Effort component. Simultaneously, students acknowledged the inherent difficulty of medical statistics learning, as indicated by a mean score of 3.49 on the Difficulty component. The mean examination score for participants stood at 72.98, aligning with the typical performance levels observed in other subjects across Chinese medical colleges, underscoring commendable learning achievements among students.

**Table 2:** SATS scores and course achievement for all the participants

Items	Mean	SD	Median
Affect	4.55	1.14	4.50
Cognitive Competence	4.63	1.12	4.50
Value	5.27	1.10	5.33
Difficulty	3.49	0.60	3.57
Interest	4.88	1.35	4.75
Effort	6.07	0.96	6.25
Achievement	72.98	12.17	74

### SATS scores and course achievement within participant subgroups

Initially, we conducted an analysis of SATS scores and exam performance across female and male student cohorts. Our examination revealed variations among the six SATS components, specifically in the components of Value, Difficulty, and Effort (Table 3). Additionally, disparities were observed in the overall examination scores. In comparison to their male counterparts, female students expressed a greater belief in the importance of statistics knowledge for their future careers. Notably, on the Effort component, female students indicated a willingness to exert more effort in medical statistics learning compared to male students. However, they also acknowledged the perceived difficulty of medical statistics more than their male counterparts did. In terms of academic achievement, female students outperformed their male counterparts, achieving a higher mean score.

**Table 3:** Comparison of SATS scores and course achievement between female and male students

Items	Female(*n* = 837)	Male(*n* = 429)	*P*-value
	Mean (SD)	Mean (SD)	
Affect	4.55(1.13)	4.55(1.17)	0.99
Cognitive Competence	4.63(1.12)	4.64(1.13)	0.81
Value	5.34(1.07)	5.15(1.17)	0.01
Difficulty	3.47(0.57)	3.54(0.65)	0.04
Interest	4.90(1.34)	4.85(1.37)	0.68
Effort	6.15(0.89)	5.91(1.08)	0.00
Achievement	74.67(11.40)	69.70(12.94)	< 0.001

Subsequently, we categorized the participants into two groups based on age, those < 21 years old and those > = 21 years. A comparison of SATS scores and examination scores between these age groups was conducted. The analysis revealed no notable variance in SATS scores between the two age groups, except for a slight discrepancy in the examination scores (Table 4). Nevertheless, given the marginal nature of the examination score difference, we deemed it lacking in practical significance.

**Table 4:** Comparison of SATS scores and course achievement between older and younger students

Items	< 21(*n* = 591)	>=21(*n* = 675)	*P*-value
	Mean (SD)	Mean (SD)	
Affect	4.59(1.14)	4.52(1.14)	0.19
Cognitive Competence	4.67(1.12)	4.59(1.11)	0.33
Value	5.30(1.10)	5.25(1.11)	0.34
Difficulty	3.48(0.58)	3.50(0.62)	0.33
Interest	4.92(1.34)	4.86(1.36)	0.55
Effort	6.14(0.91)	6.00(1.01)	0.37
Achievement	73.19(11.78)	72.80(12.50)	0.03

Finally, an examination of SATS scores and examination scores between medical undergraduates and nursing undergraduates was conducted. The analysis revealed that medical undergraduates achieved higher scores in the Affect, Cognitive Competence, and Value components compared to their nursing counterparts. Additionally, medical undergraduates outperformed nursing undergraduates in the overall examination scores (Table 5).

**Table 5:** Comparison of SATS scores and course achievement between medical and nursing undergraduates

Items	Medical undergraduates (*n* = 1188)	Nursing undergraduates (*n* = 78)	*P*-value
	Mean (SD)	Mean (SD)	
Affect	4.58(1.15)	4.14(0.92)	< 0.001
Cognitive Competence	4.65(1.12)	4.28(0.96)	< 0.01
Value	5.29(1.10)	5.01(0.88)	0.01
Difficulty	3.49(0.61)	3.45(0.52)	0.34
Interest	4.90(1.37)	4.71(1.06)	0.23
Effort	6.07(0.97)	5.96(0.86)	0.10
Achievement	73.32(11.87)	67.94(15.27)	< 0.01

### The correlation between SATS scores and the course achievement

Displayed in Table 6 are Spearman’s correlation coefficients illustrating the association between SATS scores and examination scores. Our analysis indicates a positive correlation between examination scores and the Affect, Cognitive Competence, Value, Interest, and Effort components, while a negative correlation is observed with the Difficulty component for all participants. Notably, this correlation pattern remains largely consistent across subgroups defined by gender, age, and major.

**Table 6:** Spearman’s correlation coefficients of SATS scores and learning achievements

Items	Female(*n* = 837)	Male(*n* = 429)	< 21(*n* = 591)	>=21(*n* = 675)	Medical undergraduates(*n* = 1188)	Nursing undergraduates(*n* = 78)	ALL(*n* = 1266)
Affect	0.13*	0.08	0.15*	0.08*	0.10*	0.20	0.11*
Cognitive Competence	0.18*	0.16*	0.20*	0.15*	0.16*	0.25	0.17*
Value	0.15*	0.10*	0.13*	0.16*	0.15*	0.01	0.15*
Difficulty	-0.05	-0.07	-0.02	-0.12*	-0.08*	0.02	-0.07*
Interest	0.08*	-0.02	0.06	0.03	0.04	0.05	0.04
Effort	0.16*	0.08	0.13*	0.15*	0.15*	-0.05	0.14*

*:*p* < 0.05

## Discussion

In this investigation, we explored the attitudes of medical undergraduate students toward medical statistics at a regional medical college in China. Employing SATS-36 scales, a survey was administered at the commencement of the medical statistics course. Results revealed that medical undergraduates generally harbor positive attitudes towards statistics in terms of Affect, Cognitive Competence, and Value components, while expressing negative sentiments regarding the Difficulty component of SATS-36. These findings align with those reported by previous studies [4, 8, 9, 12, 19]. Specifically, compared with Zhang et al.’s report for Chinese medical postgraduates [4], our findings suggested undergraduates exhibited a heightened affinity for medical statistics compared to postgraduates (mean of 4.55 in this study compared to 4.50). However, undergraduates demonstrated lower cognitive proficiency in learning than postgraduates (mean of 4.63 in this study compared to 4.79). Regarding the Value component, undergraduates perceived medical statistics as less valuable than postgraduates (mean of 5.27 in this study compared to 5.45). The Difficulty component, reflecting students’ perceptions of the subject’s difficulty, indicated that undergraduates perceived medical statistics as less challenging than postgraduates (mean of 3.49 in this study compared to 2.92).

We posit that compared to postgraduates, undergraduates may not fully recognize the value of statistics for their learning goals. The distinction arises from the fact that undergraduates primarily aim to pass examinations, while postgraduates must apply medical statistics for research purposes. Furthermore, as postgraduates have prior exposure to medical statistics courses during their undergraduate period, undergraduates’ relative lack of learning experiences contributes to their limited knowledge and disregard for the difficulty in learning statistics. Conversely, it could be attributed to undergraduates’ curiosity at the commencement of the course that they hold more positive feelings toward medical statistics due to their lack of prior experience. Notably, Zhang et al. did not report on Interest and Effort components. In our study, we obtained a mean of 4.88 on the Interest component, indicating students’ interest in statistics, and a mean of 6.07 on the Effort component, the highest score across all subscales, suggesting students’ willingness to invest significant effort in statistics learning despite acknowledging its difficulty.

To delve deeper into how demographic factors influence attitudes toward medical statistics and learning achievements, we scrutinized the impact of participants’ gender, age, and major. Beginning with gender, notable differences emerged in the Value, Difficulty, and Effort components. Specifically, female students exhibited a greater appreciation for statistics than their male counterparts (mean of 5.34 for females compared to 5.15 for males, *p* < 0.05). Interestingly, while females acknowledged the statistical course’s increased difficulty compared to males (mean of 3.47 for females compared to 3.54 for males, *p* < 0.05), they demonstrated a greater willingness to exert effort in their learning endeavors (mean of 6.15 for females compared to 5.91 for males, *p* < 0.05). These findings align partially with the studies conducted by Cindy van Es & Michelle M. Weaver [13], Hannigan A et al. [7], and Milic NM et al. [9]. The intriguing revelation was that female students achieved higher mean examination scores than their male counterparts (mean of 74.67 for females compared to 69.70 for males, *p* < 0.05). This observation aligns with broader education studies highlighting females’ tendency to attain higher academic achievements [20, 21]. Considering the previously mentioned attitudes of female students, we posit that their heightened recognition of the value and difficulty of statistics leads to increased attention and, subsequently, superior examination performance.

Examining age groups (< 21 vs. >=21), no differences were identified in the SATS components, consistent with the findings of Milic NM [9]. However, several studies suggest that older students typically hold negative attitudes toward statistics [4, 6, 7, 11]. Although a significant learning achievement difference was noted between age groups, the magnitude was deemed too small to hold practical significance.

Turning to the influence of majors on students’ attitudes. Participants of our study belonged to clinical medicine, dentistry, radiology, and nursing majors. Simplifying the analysis, we categorized them into medical undergraduates and nursing undergraduates. The results demonstrated medical undergraduates tended to score higher on Affect, Cognitive Competence, and Value components, achieving better course achievement compared to their nursing counterparts. In China, where the National College Entrance Examination is pivotal, nursing programs typically require lower scores than medical programs. Additionally, nursing undergraduates harbor distinct career perspectives from medical undergraduates, influencing their attitudes toward statistics and contributing to the significant learning achievement disparity observed between the two groups.

We discovered a positive correlation between course achievement and the Affect, Cognitive Competence, Value, Interest, and Effort components, while observing a negative correlation with the Difficulty component across all participant groups and subgroups. These outcomes generally align with earlier research findings, as indicated by various previous studies [22, 4, 6, 8, 9, 19]. Notably, our results specifically resonate with the findings of Nja CO et al. [19]. concerning the Difficulty component. In summary, our analysis confirmed that students exhibiting a positive attitude are inclined to achieve better learning outcomes. However, since our study was a cross-sectional survey, we couldn’t figure out whether there was a causality yet.

To summarize the previous discussion, our study found that compared with postgraduates, medical undergraduates have their characteristics on the attitude towards statistics, which manifested more positive affect but less Cognitive Competence, Value, and Difficulty components, besides confirmed gender and major factors are associated with students’ attitude towards statistics, as well as the students exhibiting a positive attitude are inclined to achieve better learning outcomes. Several studies have revealed that early learning experiences have a significant impact on the attitudes toward current statistics courses [4, 7]. Our study, along with Zhang et al. ‘s, can serve as a comparison between undergraduates and postgraduates to illustrate this conclusion. This also suggests that to improve medical postgraduates’ attitudes towards statistics, course content should be designed well during the undergraduate stage, specially making it different from the postgraduate stage, and more attention should be paid to the associated factors influencing attitude. Methods could be adding more examples of real research in accordance with students’ majors, avoiding too much mathematical theory, reducing teaching standards of calculating ability, early application of statistics analysis software, or introducing innovative pedagogical strategies. These methods require further research.

It is worth noting that our study participants were enrolled in a regional medical college in China. With a total of 304 medical colleges in the country and an estimated 120 thousand new students entering Chinese medical colleges annually, the majority typically enroll in regional medical colleges akin to the institution where our investigation was conducted. This underscores the significance of our study in contributing to the advancement of medical statistics education in China. Our findings suggest that, despite China’s strong tradition of mathematics education and students undergoing rigorous mathematical training from their primary school years, the experience of anxiety and frustration during the learning of medical statistics in college is not uncommon, which implies that teaching methods proven effective in other countries can be applied in the Chinese context.

Our study has several limitations. Firstly, it was conducted in a single medical college, and while participants hailed from diverse regions across China, the inherent biases stemming from their backgrounds could not be entirely mitigated. Secondly, the survey was administered through a web app. Although we made efforts to filter out dubious responses completed hastily, we cannot guarantee the absence of arbitrary replies from impatient participants. Thirdly, while our results affirm the effectiveness of SATS for undergraduates in China, indicating a positive correlation with medical statistics learning achievements, we did not delve into the impact of students’ backgrounds, particularly their mathematical education, which has been shown in numerous studies to influence statistics learning outcomes [4, 6, 7, 9, 23]. In subsequent investigations, we intend to explore how factors such as education, geographical location, and cultural influences contribute to medical statistics course achievement among Chinese undergraduates, aiming to uncover the key determinants in this context.

## Conclusion

The findings of this study align broadly with previous research, highlighting the difference between undergraduates’ and postgraduates’ attitudes toward medical statistics, and the influence of students’ genders and majors on their attitudes toward medical statistics. Additionally, the study reinforces the notion that students’ positive attitudes are associated with better learning outcomes. Consequently, educators should prioritize understanding the impact of attitudes, particularly considering variations related to genders and majors, when devising strategies and curricula to enhance medical statistics education for medical undergraduates.

## Data Availability

The data of this study are available from the corresponding author upon reasonable request.

## Abbreviations

SATS: Survey of Attitudes Toward Statistics
EBM: Evidence-Based Medicine
SD: Standard Deviation
R: A free software environment for statistical computing and graphics
